# Vividness, Consciousness and Mental Imagery: A Start on Connecting the Dots

**DOI:** 10.3390/brainsci10080500

**Published:** 2020-07-31

**Authors:** Amedeo D’Angiulli

**Affiliations:** Department of Neuroscience, Carleton University, Ottawa, ON K1S 5B6, Canada; amedeo.dangiulli@carleton.ca; Tel.: +1-613-520-2600 (ext. 2954)

Over twenty years ago, Baars [1] noted that, “The strategy of treating consciousness as a variable has now become standard in the study of subliminal vision, blindsight, and implicit cognition. We can easily apply it to mental imagery—yet we rarely do so”. He concluded that, “As a result, even as consciousness research thrives in other domains, we have very little firm evidence about the conscious dimension of mental imagery”. In many respects, Baar’s conclusion still stands.

Today in many studies, mental images are still either treated as conscious by definition, or as empirical operations implicit to completing some type of task, such as the measurement of reaction time in mental rotation, where an underlying mental image is assumed, but there is no direct determination of whether it is conscious or not.

The vividness of mental images is a potentially helpful construct which may be suitable, as it may correspond to consciousness or aspects of the consciousness of images. There is currently a surge of interest in vividness in cognitive neuroscience and neuroimaging literature (see [2] for a review). It seems that a general implicit assumption is that vivid images are conscious, and it is possible that the least vivid images are effectively unconscious or that they become such once a threshold (e.g., the “no image” in the Vividness of Visual Imagery Questionnaire) is reached. Thus, it is still unclear whether the vividness dimension may in fact be a kind of “disguised correlate of consciousness” [1] or if instead it might be a supramodal metacognitive dimension not necessarily associated with imagery. However, even from studies using a vividness approach in neuroscience, the conscious dimension of mental imagery is not explicitly or fully tackled head on. Indeed, when a proper exhaustive literature search is conducted, it leads back to a paper by David Marks [3] for a glimpse of what a theoretical discussion of the missing links might look like.

In this context, a complicating factor seems to be the surprising variety in what is meant by the term vividness or how it is used or theorized. Some authors do not mention imagery or consciousness at all when using the term vividness, but associate it to various forms of memory such as prospective, episodic, autobiographical, or to aliased processes not literally called imagery but, for example, imagining or visualizations or simulations. Similarly, replacement constructs for vividness have been offered, for example, in terms of the strength of imagery or semantic long-term memory contents. In all these cases, it is not really clear what is achieved by replacing one label with another or replacing a research tradition with another. We are still left with the gaps pointed out by Baars.

To start filling some of the gaps, the goal of the present Special Issue was to create a forum where authors could fully explore through sound research the missing theoretical and empirical links between vividness, consciousness, and mental imagery across disciplines.

Craver-Lemley and Reeves [4] opened this Special Issue with the study of the effect of a sweetness blocker, *Gymnema sylvestre* (a known selective suppressor of sweetness only, but not of other taste sensations), on the synesthetic taste vividness of a rare color-gustatory synesthete, E.C., for whom specific colors elicit unique tastes. Given that E.C.’s concurrent color experience can be sweet, Gymnema sylvestre provides a unique opportunity to test the role of sensory modulation in this form of synesthesia. Blocking E.C.’s sweetness receptors while the tongue was otherwise unstimulated left other taste components of the synesthesia unaltered but initially reduced her synesthetic sweetness (vividness), which suggests a peripheral modulation of the synesthetic illusion. These authors contend that although their data are from a single, very rare subject, and are speculative, this should not detract from the theoretical importance of E.C.’s synesthesia, “in which—as is otherwise almost never the case—an all-or-nothing sensory manipulation could be applied to the concurrent”. In light of this, the authors plea for the study of more cases of this sort, so that the role of an active bottom-up “tonic” signal can be further verified.

Remaining in the theme of “perceptual” vividness, Pinna and Conti [5] presented a novel take on amodal completion (AC), defined as vivid completion in a single continuous object of the visible parts of an occluded shape despite that portions of its boundary contours are not actually seen. According to their perspective, AC is a visual phenomenon and not a process (that is, not the final result of perceptual processes and grouping principles). To demonstrate this guiding proposition, the authors investigated the role of contrast polarity in newly devised stimuli assessed through the Gestalt experimental phenomenology approach. The results demonstrated the domination of the contrast polarity against good continuation, T-junctions, and regularity, even leading to the identification of a new type of junction (I-junctions). The authors defended the primacy of contrast polarity against traditional accounts of AC such as the Prägnanz principle, Helmholtz’s likelihood, and Bayesian inference.

To appreciate the difference in perspectives between the experimental phenomenology approach adopted by Pinna and Conti and the computational contenders, in this Special Issue, the readers can find a very stimulating commentary debate between these authors and the (unfortunately) late Peter van der Helm. The latter author claimed [6] that simplicity and likelihood approaches can account for the phenomena described by Pinna and Conti; and argues that Pinna and Conti’s approach erroneously confounds simplicity and likelihood. In their rebuttal, Pinna and Conti [7] rejected van der Helm’s interpretation of their hypotheses and methodological approach, noting that none of van der Helm’s comments were directly related to their stimuli. The authors clarified the complementarity of their approach with other potentially useful perspectives in explaining the yet unaccounted for stimuli they presented in the original work and their rebuttal commentary, including a possible important role of Bayesian simulations in contributing to the renewed debate on AC.

Going from perception to memory, Lefebvre and D’Angiulli [8] reported a study of the joint influences of familiarity and vividness on image generation from verbal stimuli, as characterized by image formation latency (RTs) and rate of incidental recall. Surprisingly, they found that two strong or weak codes are better than two medium ones, and that matching levels of strong or low vividness and familiarity result in faster imagery and more recalled stimuli than when both codes are of middling intensity. To explain these results, the authors proposed a dualistic neuropsychological model of memory consolidation that mimics the global activity in two large resting-state brain networks (RSNs), the default mode network (DMN) and the task-positive network (TPN). As discussed by the authors, these findings may have some applications to the clinical field for developing neurophenomenological markers of core memory deficits currently hypothesized to be shared across multiple psychopathological conditions.

Probably one of the most influential figures in vividness research, David Marks [9] took a renewed look at the systematization of the field spanning over at least four decades of work. He presented a general theory, synthesizing the reciprocal interconnections within a dynamic system involving imagery, affect, and action and consciousness as the central executive. The fundamental assumption of this general theory is that the primary motivation of all of consciousness and intentional behavior is psychological homeostasis. In this context, the degree of vividness of inner imagery is beneficial to imagining, remembering, thinking, predicting, planning, and acting. Marks reviewed key supporting work in cognitive neuroscience which directly validates introspective reports on vividness.

Thorudottir and Sigurdardottir and their team of colleagues [10] report the intriguing neuropsychological and neuroimaging case study of an architect (PL518) who lost his ability for visual imagery (aphantasia) following a bilateral posterior cerebral artery (PCA) stroke. When comparing the neuropsychological profile and structural magnetic resonance imaging (MRI) for PL518 to patients with either a comparable background (an architect) or bilateral PCA lesions, the authors found in all patients substantial shared lesions and cognitive deficits (except aphantasia, only occurring in PL518). The only selective lesions unique to PL518 were a small area in the left fusiform gyrus and part of the right lingual gyrus. These authors concluded that these areas might play a necessary role in the cerebral network involved in visual imagery.

The Special Issue concludes with an empirical examination of the vast literature. Haustein and colleagues [11] performed a bibliometric analysis of the peer-reviewed literature on vividness between 1900 and 2019 indexed by the Web of Science and compared it with the same analysis of publications on consciousness and mental imagery. While the citation patterns for papers in these three subjects are similar, the underlying topical concepts rarely overlap (co-occur) explicitly in the literature. The field of psychology dominates the topic of vividness, even though the total number of publications containing that term is small and the concept occurs in several other disciplines such as computer science and artificial intelligence. The authors suggest that without a coherent unitary framework for the use of vividness in research, important opportunities for advancing the field might be missed. The alternative is an evidence-based framework (such as bibliometric analytic methods) to guide transdisciplinary research on vividness and to resolve the challenge of conceptual, methodological, and terminological inconsistencies and differences amongst published research in multidisciplinary fields.

In conclusion, this Special Issue represented just a small cross-section of the multidimensional research landscape wherein vividness, consciousness, and mental imagery implicitly or sometimes explicitly co-exist. We are still very far from illuminating its very complex and dynamic nature rich of diverse methodological, empirical, and theoretical relationships. However, we believe we have at least planted the seeds to go a step further.
